# Relationship between the Affordances for Motor Behavior of Schoolchildren (AMBS) and Motor Competence Assessment (MCA) in Brazilian Children

**DOI:** 10.3390/children8080705

**Published:** 2021-08-16

**Authors:** Fábio Saraiva Flôres, Luis Paulo Rodrigues, Rita Cordovil

**Affiliations:** 1 KinesioLab, Research Unit in Human Movement Analysis, Instituto Piaget, Av. Jorge Peixinho 30 Quinta da Arreinela, 2805-059 Almada, Portugal; 2 Faculdade de Motricidade Humana, Universidade de Lisboa, 1499-002 Lisboa, Portugal; cordovil.rita@gmail.com; 3 Escola Superior de Desporto e Lazer de Melgaço, Instituto Politécnico de Viana do Castelo, 4900-347 Viana do Castelo, Portugal; lprodrigues@esdl.ipvc.pt; 4 Research Center in Sports Sciences, Health and Human Development (CIDESD), 5000-801 Vila Real, Portugal; 5 Interdisciplinary Center for the Study of Human Performance (CIPER), Faculdade de Motricidade Humana, Universidade de Lisboa, 1499-002 Lisboa, Portugal

**Keywords:** motor behavior, motor competence, school, schoolchildren, MCA, assessment

## Abstract

During growth, children are influenced by an extensive network, in which more favorable contexts provide better affordance landscapes, and consequently have a better potential to foster child development. We aimed to examine the affordances provided to children using the Affordances for Motor Behavior of Schoolchildren (AMBS) tool, estimating its association with children’s motor competence, as assessed by the Motor Competence Assessment (MCA) battery. Seventy-two Brazilian children were evaluated using the MCA instrument. Their parents/guardians completed the AMBS. The correlations between the two instruments (sub-scales and total scores) were investigated. ANOVAs were used to compare the motor competence performance of children with Low, Average, and High AMBS scores. Positive associations were found between AMBS and MCA, although weak to moderate in nature. In addition, children whose environments were richer in motor affordances (higher AMBS scores) showed significantly higher levels on the MCA. This study provides evidence that AMBS is a valid tool for assessing motor affordances for schoolchildren, and that those affordances are related to children’s motor competence.

## 1. Introduction

As children grow, they are influenced by an extensive network, such as their houses, the neighborhood, parents’ work, the house of relatives or friends, school, sports contexts, and culture [1]. During this process, more favorable environments, with better structural and material conditions, provide richer opportunities for action, or affordance landscapes [2], than others [3,4], having a better potential to foster child development [5]. Thus, the importance of the different environments (or microsystems) which with the child interacts during development is widely accepted in the literature [6,7,8].

A previous literature review [8] explored how studies have investigated the potential of different environments (i.e., home, school, and leisure environments) to promote children’s motor competence (MC) and motor development. The authors concluded that most studies have focused mainly on the home microsystem, providing an incomplete framework of the affordances across those environments, especially later in development. Gaps in the literature were identified concerning the study of affordances in schoolchildren’s environments and the lack of an instrument capable of analyzing the different contexts in which 6- to 10-year-old children are engaged. To address this gap, Flôres and colleagues [9] developed the Affordances for Motor Behavior of Schoolchildren (AMBS) questionnaire, which was designed to quantify motor affordances present at home and school environments. The AMBS questionnaire waw shown to represent a valuable and coherent structure of the existent affordances in three main sub-scales (Home, Materials, and School), capable of discriminating between environments according to its motor affordance enrichment. However, to be effective as an evaluation tool, the AMBS also must prove the expected relationship to children’s motor development characteristics, as their MC.

Motor competence is strongly related to the development of fundamental motor skills, comprising locomotor, stability, and manipulative skills that are cornerstones for the acquisition of specialized movements throughout the lifespan [10,11], relevant for developing children’s healthy lifestyles [10,12,13], and sport participation [14,15]. Some authors have suggested considering a broader range of movements that support physical activity engagement across the lifespan (e.g., swimming, cycling, or resistance training skills) [16,17], and the term ‘foundational movement skills’ has been proposed [18]. These foundational movement skills are influenced by socio-cultural and geographic constraints (e.g., learning to cycle might be more important to maintain an active lifestyle in countries where there is a stronger cycling culture). Despite the cultural differences, during their first years, children need to develop a motor repertoire that is sufficiently diverse to be flexibly adapted to different and specific movement contexts later in life [19]; this repertoire should always include locomotor, stability, and manipulative skills. Good levels of MC contribute to the enhanced learning of new skills and a higher motor proficiency on novel motor tasks throughout the lifespan [11]. Additionally, MC has been shown to be influenced by sex [11,20]. Studies also showed that there is a cultural influence on the levels of MC among children around the world [21,22,23], and MC has been positively associated with health-related fitness and developmental outcomes [12].

Even though MC is an extremely important topic in the study of children, a better understanding of the underlying processes that influence its development is still needed. The study of environmental influences on children’s MC has been addressed in some previous studies [24], but the availability of affordances for motor development has mostly been investigated for young children [17,25]. Given the nature of motor affordances, the construct validity of the AMBS must be validated by estimating its association with the expected output of motor affordances (i.e., motor competence). Thus, the present study aimed to examine the AMBS construct validity, estimating its association with children’s motor competence, as assessed by the Motor Competence Assessment (MCA) battery. We hypothesize that children who interact with richer motor affordance contexts (high AMBS) will present higher motor competence scores (MCA) than children living in poorer motor affordance contexts.

## 2. Materials and Methods

Two hundred and ten Brazilian families were invited (contacted using social media and schools) to participate in the present research. Seventy-two children (35 boys and 37 girls—mean age of 8.2 ± 1.4 years) and their parents or guardians agreed to enroll in this study. Participants were recruited from different cities in southern Brazil. Oral assent was obtained from the participants and written consent from their parents/guardians, before beginning the experiment. None of the participants had any developmental difficulties or medical restrictions to perform the activities. The research was approved by the university ethics committee.

From the 122 parents who agreed to participate, 103 returned the Affordances for Motor Behavior of Schoolchildren (AMBS) questionnaire [9], and 72 of their children completed the motor competence evaluation. Parents also reported their children’s age, height, and weight using the characterization category in the AMBS.

The AMBS is intended to evaluate the motor affordances provided to children by different contexts. The instrument is composed of 72 questions grouped into 11 variables (Inside Space A, Inside Space B, and Outside Space; Sedentary Material, Pretend Play Toys, Educational Toys, Manipulative Materials, and Stability Materials; Space for Movement, Free Space for Movement, and Sedentary Space), which are then organized into three main sub-scales (Home, Materials, and School). Each category raw score is transformed into a standardized score that ranges from 1 (Very Low) to 4 (Very High). The AMBS total score is made up of the sum of the three sub-scales standardized scores. In this study, children were organized into tercile groups according to the AMBS total scores, thus representing a Low (less than 8 points), Average (8 to 10 points), and High (more than 10 points) AMBS.

To assess the motor competence of the children, the Motor Competence Assessment (MCA) was administered to each child on the same day that parents completed the AMBS. Procedures are described elsewhere [11,26]. The MCA was implemented in a sports gym by three independent and fully trained investigators (Physical Education teachers), taking approximately 25 min per child.

This instrument was designed to measure motor competence and comprises six tests of three sub-scales—Stability: Jumping Sideways (JS) and Shifting Platforms (SP); Locomotor: Standing Long Jump (SLJ) and Shuttle Run (SHR), and Manipulative: Ball Kicking Velocity (BKV) and Ball Throwing Velocity (BTV). Its construct validity and normative values have been established from early childhood to young adulthood [11,27,28]. The MCA uses only quantitative and easy-to-assess tests, which diminishes observation errors, and they do not present a ceiling effect over developmental years [11,28]. The individual results (JS, SP, SLJ, SHR, BTV, and BKV) were transformed into age- and sex-related percentiles using the normative values of the MCA instrument (Rodrigues et al., 2019). To find each MCA sub-scale score (Stability, Locomotor, and Manipulative), the average of the two respective test percentile positions was used. Finally, the total MCA was calculated as the average of the three MCA sub-scales.

Descriptive analysis with mean and standard deviation was used to characterize anthropometric data and AMBS and MCA results. The Kolmogorov–Smirnov test confirmed the data normality and all statistical assumptions [29]. Pearson’s correlation was used to analyze the relationship between the MCA sub-scales and total, and the AMBS sub-scales and total. Correlation coefficients <0.30 were considered weak, those between 0.30 and 0.70 were considered moderate, and coefficients >0.70 were considered strong [29]. Univariate ANOVAs were used to find whether the AMBS classification (High, Medium, and Low AMBS) was related to MCA values (sub-scales and total). The Statistical Package for Social Sciences (SPSS), version 25.0, was used, adopting an alpha level of significance of 5%.

## 3. Results

Our results showed that regarding the income condition of the families, the answers to the AMBS showed that most of the families received less than BRL 3000 (between EUR 1501 and EUR 2500) per month (58.4%), between BRL 3001 and BRL 5000 (between EUR 2501 and EUR 3500) (19.4%), and more than BRL 5001 (over EUR 5000) (22.2%). Concerning parental education, 34.7% of the parents had failed to complete school education, 38.9% had finished high school, and 26.4% had finished higher education.

Table 1 provides information about the sample size, gender, height, weight, and extracurricular activities of the participants. In addition, it presents the data regarding MCA test percentiles, categories, and total MCA. The results showed that the children’s microsystems present low levels of AMBS Total (<8 points).

To test the associations between the AMBS and the MCA, bivariate correlations were used (Table 2). There were significant weak associations between MCA Locomotor and AMBS Materials (r = 0.232, *p* < 0.05), AMBS School (r = 0.235, *p* < 0.05), and AMBS Total (r = 0.267, *p* < 0.05); MCA Manipulative category was weakly associated with AMBS total (r = 0.279, *p* < 0.05); and Total MCA to AMBS School (r = 0.241, *p* < 0.05). Additionally, there were significant moderate associations between MCA Stability and AMBS Home (r = 0.317, *p* < 0.01), AMBS Materials (r = 0.368, *p* < 0.001), and AMBS total (r = 0.376, *p* < 0.001); MCA Manipulative category was moderately associated with AMBS Home (r = 0.313, *p* < 0.01). The Total MCA showed moderate associations with AMBS Home (r = 0.311, *p* < 0.01), AMBS Materials (r = 0.302, *p* < 0.01) and AMBS Total (r = 0.359, *p* < 0.001).

**Figure 1:**
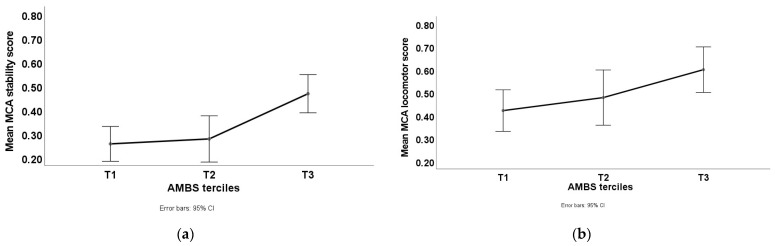

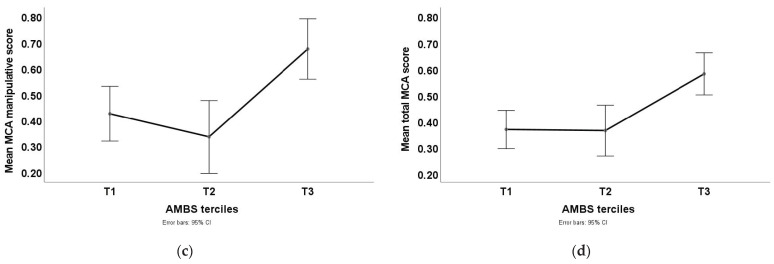
AMBS classification groups regarding MCA values: (**a**) Mean scores of the AMBS terciles in the stability component. (**b**) Mean scores of the AMBS terciles in the locomotor component. (**c**) Mean scores of the AMBS terciles in the manipulative component. (**d**) Mean scores of the AMBS terciles in Total Motor Competence.

To test for the hypothesized differences in MC according to the AMBS classification, one-way ANOVAs for each MCA sub-scale and total were performed using the three tercile groups of AMBS (Low, Average, and High). Table 3 presents the results of the main effects and post hoc tests, showing that children with AMBS higher scores presented better results in all MCA sub-scales. Children that showed a higher level of motor affordances in their environment were also significantly better on their motor competence, as shown in Figure 1.

## 4. Discussion

The purpose of the present study was to investigate the relationship between the opportunities for action available in children’s environments and their levels of motor competence. The AMBS was used to assess the quality of the different microsystems. Our initial premise was that AMBS, and MCA scores would be related, with associations between AMBS and MCA categories. Our findings confirm a significant association between AMBS and MCA categories and total scores (see Table 2). The association tested was between the opportunities for action assessed by a questionnaire and the level of MC as assessed by the MCA. The rationale was that when in the presence of more motor affordances in their daily life, children will take advantage of these opportunities, increasing their physical activity and movement experience, and with that there is the likelihood of developing a better motor competence. 

According to Gibson [2], each environment has objects, places, surfaces, events, and other people that provide different action opportunities, depending on the child’s action capabilities. This concept also shows that children experience a context according to its functionality by detecting meaningful the environmental properties of relevance to the perceiver [2,30]. Additionally, Bronfenbrenner and Ceci [7] noted that the physical, social, or symbolic environmental characteristics invite, permit, or inhibit reciprocal tuning toward a progressively more complex interactional activity in and with the immediate setting. These interactional proximal processes of development are dependent on the mutual interaction between the subject and the environment. Thus, these theoretical models, promote the understanding of MC as a result of proximal processes between a child and their immediate contexts. Powerful as it can be expected, motor affordances in the environment are not supposed to be the only influence on the motor development of children at this age. Several other features of children (personal, daily life, heredity, family, culture, society, biological development, socioeconomic condition, motivation, etc.), certainly influence children’s motor competence. Consequently, the associations found between the AMBS and the MCA, although moderate in nature, signal the important conclusion that the AMBS was able to assess and quantify important characteristics that are related to the actual development of motor competence. Thus, and even though environments are not the only influencing factor (i.e., genetic, or biological conditions are important conditions), children’s motor competence is related to the motor affordances provided in their daily life, and the AMBS is capable of capturing them.

Of the most important to this research is the insight obtained from the AMBS total scores because they consider not only the number, but also the variety of affordances. Despite the Low classification of the average number of affordances provided to children (scoring 6.28 ± 2.82)—meaning that home, materials, and schools are probably not providing all the necessary affordances to average children, school-aged children with higher affordances had significantly better levels of MC than the other two groups (see Table 3 and Figure 1). Similar results were found in several studies, showing that higher and better affordances provide higher levels of motor development in infants [31,32] and young children [33,34,35]. Thus, our study shows that affordances are extremely important to develop motor competence across the lifespan.

The AMBS proved to be an important tool to evaluate and discriminate among different motor affordance profiles. The results showed a common structured organization of potential affordances in the children’s microsystems concerning three sub-scales (Home, Materials, and School), representing a meaningful structure inside and outside the home, resulting from the parents’ decisions or possibilities on how they provide environmental stimuli to their children. Thus, our results revealed that AMBS is a valid indicator of the affordances found in multiple contexts that have the potential to influence schoolchildren’s motor competence.

Regarding the provision of affordances, other factors measured by the AMBS can contribute to the results found in the present research. For example, 58.4% of all families received less than BRL 3000 (between EUR 1501 and EUR 2500) per month, and 38.9% of the fathers and 44.4% of the mothers had only finished high school. Several studies show that financial conditions [36,37] and parental education are important aspects of child development, especially at a young age [38,39,40]. Studies have also shown that children in families with poor financial conditions, low levels of education, and huge provision restrictions present low levels of motor development [41]. In fact, school-aged children who are at risk have been shown to demonstrate developmental delays in their fundamental motor skills [42]. Thus, investigators must pay attention to these characteristics when analyzing the development of children’s MC. Some limitations of this study should be noted. First, although the AMBS is a valid and reliable assessing tool, an in loco assessment of the environments could provide better information. Secondly, the AMBS is a parental self-report instrument and provides data regarding the quantity and variety of the materials inside and outside the home; it is not purposed to assess the number of interactions that children have with the materials, nor the importance assigned to these interactions. Thirdly, affordances for fine motor skills are assessed by the AMBS, but the MCA does not assess those skills. Thus, in future studies, it might be interesting to analyze the association of AMBS with an assessment battery that also includes fine motor skills. Finally, some results in the present study might be explained by specific characteristics of our sample of southern Brazilian children, namely, the differences found between boys and girls relative to their movement activities. Our findings suggest that girls attend more team, individual and combat sports, outdoor activities, and cultural activities than boys in the same age range. These results are surprising, but the literature has shown some controversy in results when comparing boys’ and girls’ MC [21,43]. We believe that studies using AMBS to assess motor affordances provide important information that can be complemented by studies that investigate children’s actual interactions in different settings.

Future investigations could explore the relationship of AMBS with other instruments that assess different aspects of MC (e.g., fine motor skills, foundational movement skills), or with objectively measured physical activity (using accelerometers, for example). Additionally, understanding the importance of child microsystems could be important in order to devise strategies to tackle low levels of MC in school-aged children, preventing children from entering a negative spiral of MC.

## 5. Conclusions

Our findings provide further evidence that the AMBS is a valid tool for assessing motor affordances for school-aged children, being able to assess and discriminate among different motor affordance profiles. Furthermore, we can state that there is a relationship between affordances in the microsystems and children’s MC. Thus, better contextual conditions are important in the development of schoolchildren’s motor competence. Exploring the relationship of the quality and quantity of the microsystem’s affordances and its influence on the children’s motor competence development can be fundamental for understanding the complex nature of these factors, and the Affordances for Motor Behavior of Schoolchildren questionnaire proved to be a useful tool for such task.

## Figures and Tables

**Table 1:** Descriptive values of the sample.

	Boys (*n* = 35)		Girls (*n* = 37)		Total (*n* = 72)	
	Raw Scores					
	Mean	SD	Mean	SD	Mean	SD
Child Characterization						
Age	7.83	1.40	8.53	1.30	8.19	1.39
Height	118.83	21.78	119.70	23.16	119.28	22.34
Weight	31.88	8.34	30.66	9.06	31.25	8.68
Child Movement activities (days per week)						
Team Sports	0.37	0.77	1.40	1.50	0.90	1.30
Individual Sports	0.42	0.81	1.65	2.08	1.05	1.70
Combat Sports	0.0	0.0	0.38	1.11	0.19	0.82
Outdoor Activities	0.11	0.53	0.14	0.42	0.13	0.47
Music Activities	0.60	0.95	0.51	0.96	0.56	0.94
Cultural/Artistic Activities	2.54	2.10	3.24	1.82	2.90	1.98
AMBS. Sub-scales and total (raw scores)						
Home	7.11	3.15	7.32	2.33	7.22	2.75
Materials	47.94	25.60	47.62	21.83	47.78	24.09
School	9.29	3.73	9.35	4.18	9.32	3.94
AMBS total	6.23	2.97	6.32	2.74	6.28	2.83
MCA tests percentiles						
Jumping Sideways	36	27	25	27	30	28
Shifting Platforms	43	30	32	19	37	26
Standing Long Jump	56	28	58	30	57	29
Shuttle Run	46	33	40	30	43	31
Ball Throwing Velocity	62	37	42	33	52	36
Ball Kicking Velocity	49	40	44	34	46	37
MCA. Sub-scales and total (mean of test’s percentiles)						
Stability	39	23	28	19	33	22
Locomotor	50	24	49	27	49	26
Manipulative	55	35	43	28	49	32
Total MCA	48	24	40	20	44	22

MCA—Motor Competence Assessment; AMBS—Affordances for Motor Behavior of Schoolchildren.

**Table 2:** Correlation between the MCA (sub-scales and total) and AMBS (sub-scales and total).

	AMBS Sub-Scales			
MCA Sub-Scales	Home	Materials	School	AMBS Total
Stability	0.32 **	0.37 ***	0.18	0.38 ***
Locomotor	0.15	0.23 *	0.24 *	0.27 *
Manipulative	0.31 **	0.20	0.19	0.28 *
Total MCA	0.31 **	0.30 **	0.24 *	0.36 ***

MCA—Motor Competence Assessment; AMBS—Affordances for Motor Behavior of Schoolchildren; * *p* < 0.05; ** *p* < 0.01; *** *p* < 0.001.

**Table 3:** Descriptive results for each MCA sub-scale and total, according to the AMBS group classification, and ANOVAs and post hoc tests.

	AMBS Classification Groups (MCA Mean Percentiles)			
MCA	T1 (Low) (M ± SD)	T2 (Average) (M ± SD)	T3 (High) (M ± SD)	ANOVAs Post Hoc
Stability	26 ± 17	28 ± 20	47 ± 23	F (3,69) = 72.943; *p* < 0.001 T1 = T2 < T3
Locomotor	42 ± 25	48 ± 23	60 ± 26	F (3,69) = 98.014; *p* < 0.001 T1 = T2, T2 = T3, T1 < T3
Manipulative	42 ± 28	33 ± 29	67 ± 30	F (3,69) = 73.029; *p* < 0.001 T1 = T2 < T3
Total MCA	37 ± 19	36 ± 19	58 ± 23	F (2,69) = 9.252; *p* < 0.001 T1 = T2 < T3

MCA—Motor Competence Assessment; AMBS—Affordances for Motor Behavior of Schoolchildren.

## Data Availability

The data presented in this study are available on request from the corresponding author. The data are not publicly available because not all parents have given proper consent for the public display of data.
